# Extensive use of monosodium glutamate: A threat to public health?

**DOI:** 10.17179/excli2018-1092

**Published:** 2018-03-19

**Authors:** Kamal Niaz, Elizabeta Zaplatic, Jonathan Spoor

**Affiliations:** 1 Department of Pharmacology and Toxicology, Faculty of Bioscience and Agri-Food and Environmental Technology, University of Teramo-64100, Italy; 2 Faculty of Bioscience and Agri-Food and Environmental Technology, University of Teramo-64100, Italy; 3 Erasmus University Medical Centre, Erasmus University Rotterdam, Rotterdam, the Netherlands

## ⁯⁯

**Figure 1:**
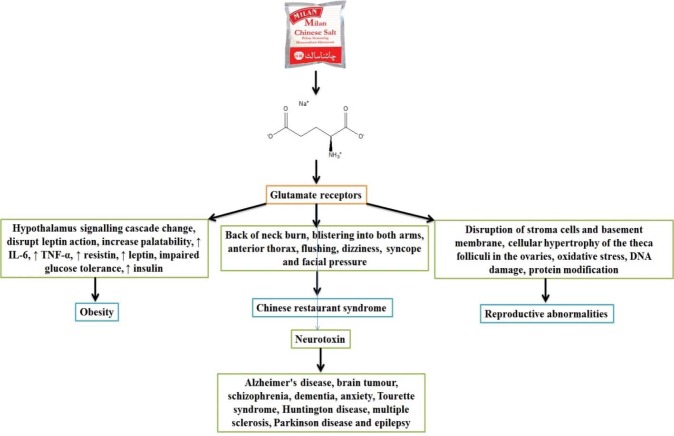
MSG toxicity leads to different disorders

Monosodium Glutamate (MSG) is one of the most widely used food-additives in commercial foods. Its application has increased over time and it is found in many different ingredients and processed foods obtainable in every market or grocery store. MSG gives a special aroma to processed foods which is known as umami in Japanese. This taste sensation is also called “savoury” (Xiong et al., 2009[19]). In many countries MSG goes by the name “China salt”. Beside its flavour enhancing effects, MSG has been associated with various forms of toxicity (Figure 1(Fig. 1)). MSG has been linked with obesity, metabolic disorders, Chinese Restaurant Syndrome, neurotoxic effects and detrimental effects on the reproductive organs. Table 1(Tab. 1) shows products containing substances that result in the release of glutamic metabolites after ingestion. The purpose of this editorial is to shed light on MSG toxicity and the possible threat it poses to public health. Can MSG associated harm be prevented or should the product be banned altogether? 

MSG acts on the glutamate receptors and releases neurotransmitters which play a vital role in normal physiological as well as pathological processes (Abdallah et al., 2014[1]). Glutamate receptors have three groups of metabotropic receptors (mGluR) and four classes of ionotropic receptors (NMDA, AMPA, delta and kainite receptors). All of these receptor types are present across the central nervous system. They are especially numerous in the hypothalamus, hippocampus and amygdala, where they control autonomic and metabolic activities (Zhu and Gouaux, 2017[22]). Results from both animal and human studies have demonstrated that administration of even the lowest dose of MSG has toxic effects. The average intake of MSG per day is estimated to be 0.3-1.0 g (Solomon et al., 2015[18]). These doses potentially disrupt neurons and might have adverse effects on behaviour. Animal studies have demonstrated that neonatal MSG consumption sets a precedent for the development of obesity later on. Insulin resistance and reduced glucose tolerance in rodents due to MSG consumption raise concerns about the development of obesity in MSG consuming humans. The same study revealed that MSG intake causes a disrupted energy balance by increasing the palatability of food and disturbing the leptin-mediated hypothalamus signalling cascade, potentially leading to obesity (Araujo et al., 2017[2]; He et al., 2011[5]). In a study into the inflammatory profile of MSG induced obesity, it has been shown that MSG triggers micro-RNA (mRNA) expression of interleukin-6 (IL-6), tumour necrosis factor-alpha (TNF-α), resistin and leptin in visceral adipose tissue. This in turn leads to enhanced insulin, resistin and leptin concentrations in the circulation and ultimately an impaired glucose tolerance (Roman‐Ramos et al., 2011[13]). In the same study, the authors were able to demonstrate that MSG induces a significant decrease in liver transaminases indicating hepatic damage. This damage was likely the result of non-alcoholic steatohepatitis which is associated with long lasting inflammation. MSG was not reported to have any effect on hunger. There are reports though of gastric distention caused by MSG two hours after ingestion. Also changes in important parameters, particularly concentrations of amino acids, have been noted. Leurine, isoleucine, valine, lysine, cysteine, alanine, tyrosin and tryptophan were significantly higher in pig blood samples after MSG consumption compared to controls. No changes have been observed in the postprandial glucose and insulin levels after intake of food supplemented with MSG (Kong et al., 2015[8]).

The term “Chinese restaurant syndrome” (CRS) was first used more than four decades ago. At the onset of symptoms patients experience complaints such as a burning sensation at the back of the neck, blistering on both arms and occasionally on the anterior thorax, general weakness, fatigue and palpitations. These symptoms occur 20 minutes after consumption of a meal rich in MSG (Bawaskar et al., 2017[3]). Other symptoms that may appear later include flushing, dizziness, syncope and facial pressure. In a study that explored negative dietary effects of MSG, double blind and placebo-controlled trails were performed in which the administration of MSG, doses ranging from 57 to 150 mg/kg, was compared with the administration of a dose of 24 mg/kg NaCl. MSG as well as NaCl administration resulted in muscle pain and/or changes in mechanical sensitivity. MSG administration however was also associated with headache and tenderness of the pericranial muscles. Furthermore, administration of a high dose of more than 75 mg/kg MSG significantly elevated systolic blood pressure (Obayashi and Nagamura, 2016[12]; Shimada et al., 2015[17]). It is not well understood if MSG is correlated with complex cases of CRS (Kazmi et al., 2017[6]). 

Both animal models and human studies have shown toxic effect of MSG on the reproductive system. Administration of MSG at a dose of 2 mg/g during various perinatal periods of life leads to an increased number of pachytene stage cells among the primary spermatocytes compared to controls in spermatogenesis (Mondal et al., 2017[10]). MSG causes disruption of stroma cell vacuolations and basement membrane- and cellular hypertrophy of the theca folliculi in the ovaries. These processes of atrophy and degeneration were assessed under different dosages (Dong and Robbins, 2015[4]). It has been well-established that MSG has some laudable gustatory and psychological effects as well as positive effects with regard to hypertension and iron deficiency. However, at the same time there are abundant reports of harmful effects such as oxidative stress, DNA damage, protein modification and lysis of stromal cells (Mustafa et al., 2017[11]).

One of the most extreme examples of negative effects attributed to MSG concerns asthma. A connection between asthma and the consumption of MSG could however never be convincingly proven. In ovalbumin-induced asthma models fed with 0.5 % and 5 % MSG, no influence was reported on eosinophil infiltration, TH-2 cytokines and immunoglobulin E (IgE) levels in the pulmonary circulation. Neither was there a measurable effect on airway hyper responsiveness (Shi et al., 2012[16]; Zhou et al., 2012[21]; Yoneda et al., 2011[20]). Injection of MSG led to bradycardia, enhanced mean blood pressure and reduced heart rate variability. It also resulted in vagal and sympathetic effect not measurable in controls (Konrad et al., 2012[9]). 

A study on a human model revealed that MSG consumption and haemoglobin levels are positively related to each other due to leptin's vital role in haematopoiesis (Shi et al., 2012[15]). Other studies have indicated however that beside MSG's stimulation, there might be other mechanisms which disrupt normal physiological function of haematopoiesis. Further research should be carried out to explore the relationship between nutritional intake of MSG and suchlike physiological mechanisms. A promising recent discovery indicates that α-ketoglutarate dehydrogenase, glutamate receptors and cysteine-glutamate antiporters have a potential role in upregulation of oxidative stress in MSG-inducted toxicity (Sharma, 2015[14]).

The harmful effects of MSG described in this paper might be perceived only by a small number of scientists, but they represent a silent threat posed by the consumption of this popular additive to all of society. It has been suggested that toxicity of MSG can be overcome by the use of certain kinds of vitamin like A, C, D and E. Quercetin and diltiazem have also been suggested to play a protective role in MSG-induced toxicity (Mustafa et al., 2017[11]). Vitamin A and C have been shown to protect nerve cells and cerebral cortex in male albino rat models. The supplementation of vitamin D and E in MSG-induced oxidative stress led to decreased lipid peroxidation, catalase and superoxide dismutase in the liver. It also improved levels of glutathione. Quercetin has been proven to reduce glucose, leptine and creatinine levels, which in turn enhances superoxide dismutase and glutathione peroxidase, while diltiazem protects against morphological functional disorders. Furthermore, new research explores the function of curcumin in the amelioration of cognitive damage via stabilisation of acetyl cholinesterase (AchE) levels and reduction of TNF-α. Furthermore, curcumin acts as a protective agent against neural damage due to its effect of decreasing the expression of mGLUR5 and *N*-Methyl-*D*-aspartate receptors 2B (NMDA2B) in the hippocampus. Because of its properties that help balance glutamate levels scientists have suggested the introduction of combinations of curcumin and MSG in the market (Khalil and Khedr, 2016[7]).

In conclusion we would like to state that although MSG has proven its value as an enhancer of flavour, different studies have hinted at possible toxic effects related to this popular food-additive. These toxic effects include CNS disorder, obesity, disruptions in adipose tissue physiology, hepatic damage, CRS and reproductive malfunctions. These threats might have hitherto been underestimated. In the meantime, people keep using ever larger amounts of MSG unaware of the possible consequences. Further studies need to be undertaken in order to assess the connection between MSG and cardiovascular disorders, headache, and hypertension in human models. MSG is a controversial food-additive used in canned food, crackers, meat, salad dressings, frozen dinners and a myriad of other products. It is found in local supermarkets, restaurants and school cafeterias alike. While MSG probably has huge benefits to the food industry, the ubiquitous use of this food-additive could have negative consequences for public health. If more substantive evidence of MSG-toxicity would be provided, a total ban on the use of MSG as a flavour enhancer would not be unwise to consider.

## Conflict of interest

There is no conflict of interest.

## Acknowledgements

The authors who worked on this manuscript acknowledge their respective universities and institutes.

## Figures and Tables

**Table 1:**
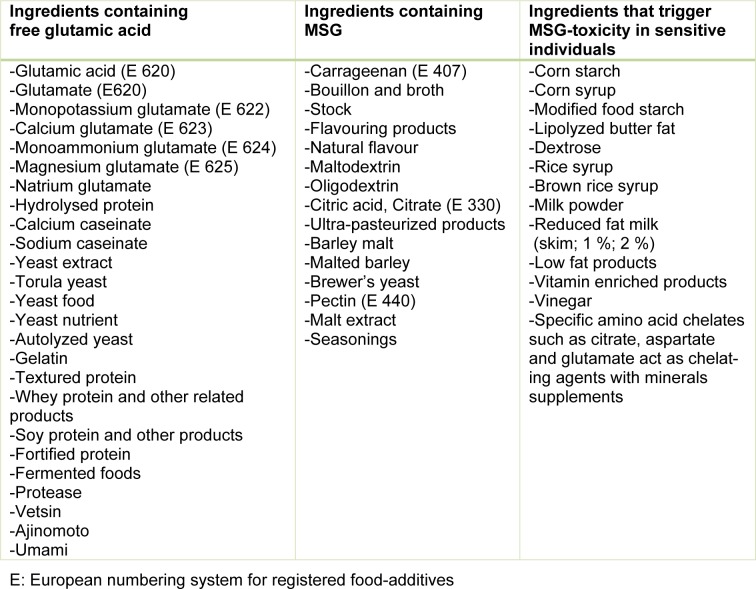
Different sources of MSG in commercial products
